# The Effect of Pain Coping Strategies on Perceived Social Support and Acceptance of Pain in Elderly Individuals With Chronic Pain

**DOI:** 10.1155/prm/6417337

**Published:** 2025-02-24

**Authors:** Zahra Eshkevar-Faraji, Zahra Fotokian, Zahra Jannat Alipour, Ali Pourhabib

**Affiliations:** ^1^Student Research Committee, Nursing Care Research Center, Health Research Institute, Babol University of Medical Sciences, Babol, Iran; ^2^Nursing Care Research Center, Health Research Institute, Babol University of Medical Sciences, Babol, Iran

**Keywords:** chronic pain, coping strategies, older adult, pain acceptance, perceived social support

## Abstract

**Background and Objective:** Chronic pain represents not only an unpleasant physical condition but also numerous psychological and social consequences for older adults, potentially diminishing their quality of life. Gaining insight into the connection of pain coping mechanisms with pain acceptance and perceived social support can facilitate the development of effective approaches for the treatment and management of pain in older adults. The present study was conducted with the aim of determining the effect of pain coping strategies on perceived social support and pain acceptance in older adults with chronic pain.

**Methods:** The current research was a descriptive, analytical, and correlational study. Participants were selected by a simple random method and comprised 363 older adults with chronic pain referred to the specialized clinics of selected medical centers in the west of Mazandaran province. Tools used to collect data included the Multidimensional Scale of Perceived Social Support (MSPSS), the chronic pain acceptance instrument in older adults (ECPAI), the pain coping strategies questionnaire (PCSQ), and the VanKroff Graded Chronic Pain Scale (VGCPS).

**Results:** The average age of the participants was 68.18 ± 6.36 years. Based on the results of the Pearson correlation test, a positive and significant relationship was found between pain coping strategies (except catastrophizing) and perceived social support (*p* < 0.001). The highest correlation with perceived social support was observed in components of faith and praying, hoping, and ignoring the pain, with a coefficient of 0.35. Moreover, there was a positive and significant relationship between acceptance of pain and reinterpreting pain, return attention, talking to oneself, ignoring the pain, distractor behaviors, praying, and hoping (*p* < 0.001). Praying and hoping components exhibited the strongest correlation with pain acceptance, with a coefficient of 0.32.

**Conclusion:** The results showed that coping strategies influence pain acceptance and perceived social support among older adults with chronic pain. Therefore, it is suggested that health service providers, especially nurses, implement appropriate educational, care, support, and psychological solutions in order to empower older adults to recognize and apply effective and efficient coping strategies.

## 1. Introduction

During recent decades, there have been significant changes in the age distribution of populations, particularly that of the developing countries. According to a report by the World Health Organization (WHO), the number of individuals aged 65 and above will double in the next 40 years, with 52% residing in Asian countries and 40% in developed nations [1]. Statistical data from Iran indicate that by 2050, older adults will account for more than 20% of the country's total population [2].

Older adults face numerous challenges associated with old age [3]. One prominent health issue among this population is chronic pain [4], which is characterized by pain that lasts for at least 3 months of the past 6 months or persistent pain that extends beyond 3 months [5]. The estimated prevalence of chronic pain among older adults in various societies ranges from 54% to 70% [6]. In Iran, the prevalence of chronic pain among individuals aged 60–90 has been reported to be approximately 67% [7].

Not only does chronic pain significantly impact the physical and mental capabilities of older adults [8], but it also leads to various social restrictions [9], depression [10], anxiety [11], sleep disorders [12], feelings of loneliness [13], and diminished independence [14]. Consequently, these factors contribute to a marked decline in the overall quality of life for older adults. In this context, the capacity of older individuals to manage and cope with pain is crucial for enhancing their mental and social well-being, as well as their quality of life [15]. A key element influencing how older adults confront chronic pain is the coping strategies they adopt [16].

Coping strategies encompass a set of cognitive and behavioral efforts that are used by an individual to interpret and manage a stressful situation and cope with problems. These strategies play an essential role in both physical and mental health [17]. Coping with pain involves specific thoughts and behaviors aimed at managing pain and emotional responses to pain, ultimately improving adaptation and preventing chronic pain development [18].

Specifically, coping strategies refer to the methods individuals employ to manage pain and the associated challenges [18]. These strategies can be categorized into two primary types: active and passive coping strategies. Active coping strategies involve direct actions taken by individuals to reduce pain, where they play an active role in pain management. Such strategies typically include individual efforts to alleviate or control pain through various physical, cognitive, or emotional approaches. Individuals actively seek out solutions, information, or techniques that may assist in pain reduction or management. These strategies are often associated with efforts to enhance quality of life and acceptance of pain, which help promote a sense of control and personal empowerment. Common active coping strategies include problem-focused coping, creating a positive mindset, engaging in physical activity and exercise, and employing relaxation and deep breathing techniques. Conversely, passive coping strategies are characterized by behaviors where individuals do not actively engage in pain reduction and tend to avoid or limit physical and social activities. Such strategies may involve restricting activities, evading pain, ignoring it, or depending on others for pain relief. While these passive strategies may provide short-term relief, they can lead to long-term psychological and physical issues, such as depression and heightened feelings of helplessness, ultimately reinforcing a sense of inadequacy and dependence on others, thereby diminishing the overall quality of life [19].

Pain acceptance and social support are two critical factors that, in conjunction with coping strategies, significantly influence the experience of pain and its management. Accepting pain does not imply surrendering; rather, it involves acknowledging the presence of pain as an experience, rather than attempting to deny, control, or eradicate it, and refraining from perceiving it as a threat to all aspects of life. From a psychological standpoint, pain acceptance enables individuals to manage their pain more effectively and with reduced stress [20]. Furthermore, it can enhance the quality of life for those experiencing chronic pain by alleviating anxiety, depression, and physical limitations [21]. Nevertheless, research indicates that pain acceptance necessitates both psychological and social support to ensure individuals can navigate this process successfully and reap its benefits. Indeed, insufficient social support may intensify the pain experience and elevate anxiety and depression levels among individuals with chronic pain [22]. Social support serves as a crucial psychological asset that significantly aids individuals in managing pain, particularly in instances where they experience loneliness and a lack of control over their pain. Perceived social support refers to the subjective sense of belonging, acceptance, love, and affection, in addition to the perceived level of assistance they believe they receive from others [23]. Perceived social support can significantly influence emotional regulation and alleviate pain-related stress and anxiety in those experiencing chronic pain. This is particularly crucial when pain is a constant presence in daily life, necessitating access to supportive resources to address the psychological and social challenges it presents. Support from family, friends, and community groups can mitigate feelings of loneliness and isolation, fostering a sense of belonging and psychological safety. Such support can also help individuals diminish negative emotions related to pain and enhance their coping mechanisms [24]. The nature and sources of social support are influenced by the cultural, social, and economic contexts of different societies. Nonetheless, the perception of older adults regarding the type and extent of support they receive is of paramount importance [25]. Indeed, how older adults perceive the support available to them determines whether they view it as positive or negative [26].

Research findings indicate a correlation between the coping strategies employed by patients with chronic pain and levels of pain acceptance and social support. McCracken and Vowles [22] demonstrated that individuals who engage in more active coping strategies, such as cognitive coping, are more likely to exhibit pain acceptance [22]. The findings of a study by Vowles, Sowden, and Ashworth [20] demonstrated that patients on cognitive–behavioral therapies reported significantly greater pain acceptance compared to those who relied on avoidant coping strategies. Furthermore, individuals who adopted active coping strategies had better social relationships and received greater support [27]. In another study, de C Williams et al. [28] showed that cognitive coping serves as a predictive factor for pain acceptance among chronic pain patients [28]. Additionally, Cohen, Janicki-Deverts, and Miller [29] demonstrated that individuals employing avoidant coping strategies are more inclined to distance themselves from their support networks and possess a diminished perception of social support, often feeling that others are unable to adequately understand or assist them [29]. Gong et al. [30] found that patients with chronic pain who utilized more active and social coping strategies experienced significantly enhanced social support [30].

Current findings indicate that coping strategies are correlated with pain acceptance and social support; however, the experience of pain, the degree of acceptance and coping, and perceived social support are significantly shaped by individual, cultural, and social influences. Consequently, variations in these factors can yield differing outcomes in cross-cultural studies. Thus, conducting research across diverse cultural contexts may enhance the validity and generalizability of the findings. Given that older adults are particularly vulnerable to chronic pain and its associated consequences due to the high incidence of multiple chronic conditions and physiological changes [31], it is crucial to implement comprehensive and multidimensional pain management models tailored to their needs. These models should encompass not only the physiological aspects of pain but also the psychological, social, cultural, and individual factors. They must utilize all available resources, including coping mechanisms, pain acceptance, and social support, to assist individuals in managing chronic pain [32]. Nevertheless, current research focusing on chronic pain among older adults has largely treated these categories in isolation, with limited studies exploring the concurrent interconnections among coping strategies, pain acceptance, and perceived social support. Consequently, there is a clear necessity for additional research to investigate the interactions between these components, particularly across diverse cultural and social contexts. Such investigations could lead to the development of more effective interventions and management approaches for chronic pain, ultimately enhancing the quality of life for older adults. Therefore, this study aimed at exploring the effect of pain coping strategies on perceived social support and pain acceptance in older adults with chronic pain.

## 2. Materials and Methods

This descriptive, analytical, and correlational research was conducted during the years 2022–2023. The study population comprised older adults with chronic pain referred to specialized clinics of selected medical centers in the west of Mazandaran province, Iran. The sample size was calculated using the formula for estimating the population mean *n*=*s*^۲^(*Z*_1−*α*/2_+*Z*_1−*β*_)^۲^/*d*^۲^ and the results of a pilot study on 10 individuals who had primary roles as older adults with chronic pain. Considering an alpha of 0.05, *Z*_1−*β*_=0.84, *S* = 0.91, and effect size *d* = 20% of standard deviation, an estimated 330 subjects were needed for this study. A sample size of 363 was calculated, taking into account a 10% attrition rate. Sampling was carried out using a simple random method. Inclusion criteria comprised age of 60 years or older, willingness to participate in the study, ability to read and write, absence of significant vision and hearing impairments hindering communication, presence of chronic pain (persistent or recurrent for longer than 3 months), absence of malignant diseases or mental disorders (as documented in the medical records), and absence of cognitive disorders (scoring 7 or higher in the Persian version of the Short Cognitive Status Test). Exclusion criteria comprised unfinished surveys. The Persian versions of the following tools were utilized to collect data:

A. Demographic clinical profile: Patient profiles consisted of various parameters including age, sex, marital status, number of offspring, education level, income status, occupational status, insurance, place of residence, living status, history of underlying disease, duration of disease, duration of pain, and location of pain.
B. Multidimensional Scale of Perceived Social Support (MSPSS): Developed by Zimmet et al., this questionnaire comprises 12 items measured on a five-point Likert scale ranging from 1 (Completely Disagree) to 5 (Completely Agree). This scale provides a general score as well as specific scores for family support, support from friends, and support from other significant individuals. The score ranges from 4–20. The level of perceived social support is considered low for scores of 12–20, moderate for 20–40, and high for scores of more than 40. Zimmet et al. reported the Cronbach's alpha coefficient of this scale to be between 0.85–0.91 and 0.72–0.85 with the retest method [33]. Bagherian-Sararoudi et al. [34] reported the reliability of the questionnaire to be 0.83% by Cronbach's alpha method [34]. In this study, the reliability of the instrument was evaluated using the internal consistency and consistency reliability method. The internal consistency of the questionnaire was confirmed with Cronbach's alpha (0.95), and the reliability was confirmed with the intraclass correlation coefficient (0.98).
C. Chronic Pain Acceptance Instrument in older adults: Developed by Shirazi et al. [35], this seven-item tool assesses the acceptance of chronic pain in older individuals. The internal consistency of the questionnaire was confirmed with a Cronbach's alpha of 0.83 and a retest reliability of 0.85. This questionnaire explores the concept of acceptance of chronic pain in older adults via two dimensions of awareness about pain (two items) and efforts to reduce pain (five items). Responses are rated on a five-point Likert scale: Always (4 points), Often (3 points), Sometimes (2 points), Rarely (1 point), and Never (0 points), with total scores ranging from 0 to 28. Higher scores indicate a greater level of acceptance of chronic pain in older adults [35]. This study used internal consistency and stability reliability to evaluate the reliability of the instrument. The internal consistency of the questionnaire was confirmed with a Cronbach's alpha of 0.72 and the stability reliability with an intraclass correlation of 0.96.
D. Rosenstein and Kiev pain coping strategies: This 42-item scale was developed by Rosenstein and Kiev in 1983. Coping strategies include six cognitive strategies (return attention, reinterpreting pain, talking to oneself, ignoring the pain, catastrophizing, and praying and hoping) and one behavioral strategy (increased behavioral activity). Each coping strategy consists of six items graded on a seven-point Likert scale. The total score of the each coping strategy lies in the range of 0–36. A higher score in each strategy indicates more use of that strategy when facing pain [36]. In Iran, the psychometric properties of the questionnaire were investigated by Asghari Moghadam and Golak [37], and the Cronbach's alpha coefficient was calculated for each of the strategies: return attention (*α* = 0.82), reinterpretation pain (*α* = 0.77), talking to oneself (*α* = 0.82), ignoring pain (*α* = 0.83), praying and hoping (*α* = 0.74), increased behavioral activity (*α* = 0.75), and 0.926 for the whole questionnaire [37]. In this study, the reliability of the instrument (by the method of internal consistency and consistency reliability) was done. The internal consistency of the questionnaire was confirmed with a Cronbach's alpha of 0.93 and the consistency reliability with an intraclass correlation of 0.98.
E. VanKroff Graded Chronic Pain Scale (VGCPS): This tool was developed by Van Kroff et al., in 1992, with the aim to assess the severity of chronic pain. This questionnaire evaluates three aspects of pain intensity, persistence or duration of pain, and the degree of disability caused by pain. Respondents rate each of the seven questions on an 11-point scale ranging from 0 to 10. The total scores on this questionnaire range from 0 to 70. Scores between 0 and 20 denote Low Chronic Pain, scores between 20 and 35 represent Moderate Chronic Pain, and scores above 35 indicate Severe Pain. The reliability of this questionnaire has been established with a Cronbach's alpha of 0.90 [38]. In a study conducted by Alilou, Pak, and Alilou, the reliability of the questionnaire was reported to be 0.83 using Cronbach's alpha [39]. In this study, the reliability of the tool was assessed via internal consistency and stability reliability methods. The internal consistency of the questionnaire was confirmed with a Cronbach's alpha of 0.94, and the stability reliability of the instrument was confirmed with an intraclass correlation of 0.98.

The researcher visited the clinics on the working days and hours, and a list of eligible older adults was prepared based on predetermined criteria and placed in the sampling frame. A random selection of samples was subsequently made. After explaining the research objectives and obtaining informed consent, the researcher collected data by providing the research tools to participants. The participants were given a verbal explanation on how to complete the questionnaires and were asked to fill them out. The questionnaires were completed by older adults in a designated room within the clinic. There were a considerable number of participants enrolled in the study, and thus, this process was performed over multiple sessions. Due to the need to fill out more than one questionnaire and the physical and mental limitations of participants, those who were unable to complete the questionnaires at the clinic were given the option to take them home. To facilitate the return of the completed questionnaires, the researcher obtained the contact information of older adults or their caregivers. This allowed the researcher to collect the questionnaires as agreed upon. Additionally, the researcher provided their contact details in writing to older adults or their caregivers for any questions or clarifications on questionnaire completion. It is important to note that the number of samples collected per sampling day ranged from 5 to 15 individuals.

SPSS software (Version 26) was used to conduct data analysis, employing both descriptive and inferential statistics. The initial step in quantitative data analysis within the inferential statistics evaluations involved examining the normality of the data. All variables exhibited normal distribution. Consequently, various statistical tests such as independent *t*-tests, one-way analysis of variance, Pearson's correlation coefficient, and multiple regression were employed. The tests were conducted with a significance level set at less than 0.05.

## 3. Findings

The study participants included 343 older adults experiencing chronic pain. The average age of the participants was 68.18 ± 6.36 years, falling within the age range of 60–94 years. The results indicated that the majority of the study population consisted of 185 females (*n* = 158, 53.9%), and married (*n* = 296, 86.3%), with moderate income (*n* = 280, 81.6%). The most common cause of pain among older adults was arthritis, reported by 168 individuals (48.98%). Additionally, 297 participants (86.6%) had an underlying medical condition. The average duration of chronic pain among older adults under investigation was 10.42 ± 7.54 years. Furthermore, a significant proportion of participants (72.6%) reported experiencing pain in more than two sites (Table 1).

According to the findings, the mean chronic pain experienced by the participants was 38.18 ± 11.25, indicating a high level of pain. The number of participants with severe, moderate, and low levels of pain was 197 (57.4%), 131 (38.2%), and 15 (4.4%), respectively. In terms of pain acceptance, the average score was 19.83 ± 4.33, indicating a high level. A total of 308 participants (89.8%) reported high levels of pain acceptance, while 35 individuals (10.2%) reported low levels. Furthermore, the research findings revealed a high level of average perceived social support among older adults with a score of 44.25 ± 5.55. In terms of coping strategies employed by the participants when in pain, the lowest average score pertained to the reinterpretation of pain (14.44 ± 7.28), while the highest average score (23.91 ± 5.53) was observed for praying and hoping (Table 2).

The Pearson correlation coefficient was utilized to explore the association between pain coping strategies, pain acceptance, and perceived social support among older adult individuals suffering from chronic pain. Prior to conducting this analysis, the assumption of normality was assessed by examining skewness and Kurtosis values. The skewness and Kurtosis values for pain coping strategies, pain acceptance, and perceived social support fell within the acceptable range of ±2, confirming the normality assumption for the research variables. The findings of the Pearson correlation analysis revealed a significant positive relationship between pain coping strategies (excluding catastrophizing) and perceived social support in older adults with chronic pain (*p* < 0.001). Specifically, the components of praying, hoping, and ignoring pain exhibited the highest correlation with perceived social support, with a coefficient of 0.35 (Table 3).

The results of Pearson's correlation test showed a positive and significant relationship between the coping strategies of reinterpreting pain, return attention, talking to oneself, ignoring pain, increasing behavioral activity, and praying and hoping with acceptance of pain (*p* < 0.001). In addition, a negative and significant relationship was demonstrated between the coping strategy of catastrophizing pain and acceptance of pain (*p*=0.021). The praying and hoping component had the highest correlation with acceptance of pain with a coefficient of 0.32 (Table 4).

To investigate the effect of pain coping strategies on perceived social support in the study participants, multiple regression was used in a stepwise manner. Independent variables (demographic variables and pain coping strategies) were included in three models. Ignoring the pain was entered in the first model and explained 11% of the variation in perceived social support. In models two and three, praying and hoping, and living status were entered into the model and finally explained 17.8% of the changes in perceived social support (Table 5).

Praying and hoping exhibited the strongest impact on perceived social support, as indicated by a standardized beta coefficient of 0.243. This implies that a one standard deviation increase in praying and hoping leads to a 0.243 unit rise in the perceived social support experienced by older individuals. Following praying and hoping, ignoring the pain demonstrated the most significant regression effect on perceived social support, with a standard beta coefficient of 0.225. This suggests that a one standard deviation increase in the ability to ignore pain results in a 0.225 unit increase in perceived social support among older adults. Conversely, living with a family member exerted an influence on the perceived social support of older individuals showing a standard beta coefficient of −0.150. This signifies that the level of perceived social support among older adults living alone was 0.150 units lower compared to their counterparts.

In order to examine the impact of pain coping strategies on pain acceptance among older adults suffering from chronic pain, a stepwise multiple regression analysis was conducted. Four models were developed, incorporating independent variables such as demographic factors and pain coping strategies. The initial model included praying and hoping, which accounted for 10% of the variance in pain acceptance. Subsequent models incorporated variables related to increased behavioral activity, pain catastrophizing, and the number of pain sites, ultimately explaining 16.3% of the variance in pain acceptance. Among the variables, praying and hoping exhibited the highest regression effect on pain acceptance, with a standard beta coefficient of 0.223. This suggests that for every one standard deviation increase in praying and hoping, there is a 0.223 unit increase in pain acceptance among older adults. Following praying and hoping, increased behavioral activity, pain catastrophizing, and the number of pain sites had regression effects on pain acceptance, with the standard beta coefficients of 0.191, −0.170, and −0.137, respectively. This indicates that a one standard deviation increase in the behavioral activity leads to a 0.191 unit increase in pain acceptance, while the same increase in pain catastrophizing and number of pain sites results in a 0.170 and 0.137 unit decrease in pain acceptance among older adults (Table 6).

## 4. Discussion

The results of this study demonstrated a significant and positive association between pain coping strategies and pain acceptance (excluding catastrophizing) among older adults with chronic pain (*p* < 0.001). Notably, the praying and hoping component exhibited the strongest correlation with pain acceptance, *d*, a coefficient of 0.32.

This outcome is consistent with the findings of previous studies conducted by Alilou, Pak, and Alilou [39], Rad and Rafezi [40], Droppert and Knowles [41], McCracken and Vowles [22], and de C Williams et al. [22, 28]. The study by Rad and Rafezi revealed a significant negative relationship between emotion-focused strategies and avoidance strategies with pain acceptance, while a positive and significant relationship was observed between problem-oriented strategies and pain acceptance [40]. Consequently, decreasing emotion-focused and avoidance strategies, while enhancing problem-oriented strategies, led to higher pain acceptance scores in patients with rheumatoid arthritis. A study by Droppert and Knowles [41] demonstrated that adoption of adaptive coping strategies was significantly associated with pain acceptance. Accordingly, the utilization of adaptive coping strategies results in increased pain acceptance among fibromyalgia patients [41]. Furthermore, Mahmoud Alilou's study [39] indicated that coping strategies play a mediating role in pain acceptance [39]. Notably, we did not encounter any studies in the literature review that contradicted the findings of the present study.

A study conducted by McCracken and Vowles [22] revealed that individuals employing more active coping strategies, including cognitive coping, demonstrated a higher level of pain acceptance [22]. Additionally, de C Williams et al. [28] found in their research that cognitive coping serves as a predictive factor for pain acceptance among patients suffering from chronic pain [28].

Consistent with the findings of this study, the research conducted by Rad and Rafezi [40] indicated that the use of avoidance or negative emotion-focused strategies such as catastrophizing may hinder pain acceptance and lead to unsuccessful pain management. Individuals experiencing more severe pain tend to resort to maladaptive coping strategies, whereas those utilizing adaptive and positive strategies are better equipped to handle their pain. It appears that individuals who have not acquired adaptive and positive coping mechanisms may struggle with accepting and managing their pain. Furthermore, emotion-focused coping strategies and positive adaptation, which focus on diminishing negative emotions stemming from pain, can influence the level of pain acceptance among individuals with chronic pain [42].

Active coping strategies appear to assist individuals in managing pain and accepting it as an experience in life. Such strategies encourage individuals to perceive pain as a normal and manageable challenge, thereby enhancing their acceptance. Conversely, passive strategies, including avoidance or denial of pain, can result in negative emotions and hinder the individual's ability to accept pain as an aspect of their life [43].

Individuals who employ positive and efficient coping mechanisms, such as engaging in praying and hoping, return attention, engaging in talking to oneself, reinterpreting pain, ignoring pain, and increasing their behavioral activity, experience a heightened sense of intrapsychic cohesion and integration. As a result, stressors such as pain are perceived as manageable issues. Conversely, individuals who utilize negative and ineffective emotional strategies, such as catastrophizing, tend to feel more disconnected and helpless. They view stressors associated with illness, including pain, as threatening and catastrophic events [43]. Research findings indicate that patients who exhibit higher levels of pain acceptance demonstrate greater compatibility with chronic pain [44, 45]. Therefore, it is crucial to encourage the use of effective and appropriate coping strategies among older adults, enabling them to accept chronic pain to the greatest extent possible. This approach can ultimately contribute to improved pain management.

The findings of this study indicated a significant and positive correlation between pain coping strategies (excluding catastrophizing) and perceived social support among older adult individuals with chronic pain (*p* < 0.001). Specifically, praying, hoping, and ignoring pain demonstrated a coefficient of 0.35 and exhibited the strongest correlation with perceived social support. In other words, engaging in praying, hoping, and ignoring pain strategies was associated with higher levels of perceived social support. This is consistent with the results of previous research conducted by López-Martínez, Esteve-Zarazaga, and Ramírez-Maestre, which demonstrated a direct relationship between active (adaptive) coping strategies and perceived social support in chronic pain patients [46].

The findings of a study conducted by Vowles, Sowden, and Ashworth [20] indicated that individuals employing active coping strategies experienced enhanced social relationships and greater support [27]. The investigation by Cohen, Janicki-Deverts, and Miller [29] revealed that those utilizing avoidant (passive) coping strategies had a diminished perception of social support [29]. Additionally, Gong et al. demonstrated that individuals who engaged in more active and social coping strategies received significantly more social support [30]. These results align with the conclusions drawn from the current study.

Additionally, the study by Roohafza et al. revealed higher levels of perceived social support in individuals utilizing problem-focused coping strategies (active coping strategies) compared with those employing emotional and avoidance coping strategies [47]. It appears that effective coping mechanisms such as praying and hoping not only provide individuals with spiritual strength to endure hardships but also lead to positive psychological outcomes such as finding meaning in life, fostering hope, reducing feelings of helplessness and loneliness, decreasing disability, and instilling a sense of vitality by dispelling negative emotions, negative thoughts, fear, and anxiety.

Individuals who manage their pain through active coping strategies tend to establish stronger connections with others and utilize social resources more effectively. Such individuals frequently seek assistance from others in addressing challenges and openly share their emotions.

Our results also demonstrated that perceived social support was most influenced by praying and hoping, as evidenced by the highest regression effect. Additionally, ignoring pain was found to have the greatest regression effect on perceived social support following engagement in praying and hoping. Moreover, the variable of living status had an impact on the perceived social support of older individuals. Our review of the literature did not uncover any similar studies that examined the influence of coping strategies on perceived social support. Consequently, this section discusses some studies conducted on the relationship between pain coping strategies and other variables. Rezaei et al. [48] discovered that pain catastrophizing and talking to oneself were the coping strategies with the most significant regression effect on depression in chronic pain patients [48]. Similarly, Davoudi et al. found that reinterpreting pain had the highest regression effect on functional disability in patients with rheumatism [49]. Hence, coping strategies impact the perception of pain intensity, the ability to control and tolerate pain, and the continuation of daily activities. Studies have demonstrated that active strategies, such as engaging in activities despite the pain, ignoring the pain, and utilizing relaxation techniques, contribute to better adaptation and pain management. Conversely, passive strategies such as catastrophizing, dependence, and limiting activities are associated with increased pain, more severe physical disability, heightened fear, increased risk of depression and anxiety, and feeling of loneliness [39, 40]. In such circumstances, psychological skills are likely to diminish, leading to a weakened perception of social support.

We also found that pain acceptance was most significantly influenced by praying and hoping. Furthermore, the acceptance of pain in older adults was primarily influenced by faith and hope followed by increased behavioral activity, pain catastrophizing, and the number of pain sites. Rad and Rafezi study revealed that emotional coping strategies, avoidance coping strategies, and problem-oriented coping strategies had the greatest regression effect on pain acceptance [40]. It appears that the utilization of appropriate coping mechanisms enhances coping efficacy, enabling individuals to have more control over pain and effectively reduce it. Efficient management of pain enables individuals to continue engaging in daily activities despite its presence, while simultaneously exerting greater effort to control the pain. This adaptive response signifies the acceptance of pain and the ability to adapt to the changes it brings.

One of the limitations of the recent research lies in its cross-sectional design, which solely investigates relationships at a single point in time and fails to capture changes over a period. Additionally, the study employed self-reported questionnaires, which may be associated with inaccuracies in responses due to reasons such as fear of judgment, the inclination to present oneself favorably, or lapses in memory. These factors may compromise the precision and reliability of the findings. Consequently, it is recommended that future studies take these limitations into account.

## 5. Conclusion

The findings of this study indicated that employing adaptive and efficient strategies can enhance pain acceptance and increase perceived social support in older adults with chronic pain. Given that pain acceptance and perceived social support are recognized as significant psychological factors in adapting to pain, the findings of this research may assist healthcare professionals, particularly nurses, in developing innovative care models for older adult individuals experiencing chronic pain. These care models should aim to empower older adults to adopt suitable and effective coping mechanisms, promote pain acceptance, and enhance perceived social support, ultimately resulting in improved adaptation to chronic pain.

## Figures and Tables

**Table 1:** Demographic characteristics of study participants.

Demographic variable			
Gender	Male	158	46.1
	Female	185	53.9

Marital status	Single	10	2.9
	Married	296	86.3
	Widow	37	10.8

Number of offspring	No children	15	4.4
	1–3 children	216	63
	More than 3 children	112	32.6

Income status	Low	42	12.2
	Moderate	280	81.6
	High	21	6.1

Education	Illiterate	19	5.5
	Elementary school	94	27.4
	Middle school	89	25.9
	High school diploma	92	26.8
	Higher than diploma	49	14.3

Number of pain areas	Two or less	94	27.4
	More than two	248	72.6

Age (years)	Standard deviation ± mean	68.18 ± 6.36	
	Minimum–maximum	60–94	

Employment status	Employed	112	32.6
	Not employed	231	67.4

Insurance status	No	29	8.5
	Yes	314	91.5

Supplementary insurance	No	174	50.7
	Yes	169	49.3

Living status	With spouse	129	37.6
	With spouse and children	158	46.1
	With children	24	7
	Single	32	9.3

Chief problem associated with chronic pain	Spinal disc	100	29.15
	Arthritis	168	48.98
	Rheumatoid arthritis	25	7.29
	Neuropathy, vascular problems	50	14.58

Underlying disease	No	46	13.4
	Yes	297	86.6

Duration of illness (years)	Standard deviation ± mean	11.62 ± 9.70	
	Minimum–maximum	3–34	

Duration of chronic pain (years)	Standard deviation ± mean	10.42 ± 7.54	
	Minimum–maximum	1–18	

**Table 2:** Pain coping strategies in older adults with chronic pain.

Pain coping strategies	Mean (standard deviation)	Maximum–minimum	Level⁣^∗^	
			Low *n* (%)	High *n* (%)
Catastrophizing	18.38 (6.56)	36–1	161 (46.9)	182 (53.1)
Reinterpretation	14.44 (7.28)	33–0	211 (61.5)	97 (28.3)
Return attention	19.54 (7.34)	35–2	146 (42.6)	197 (57.4)
Talking to oneself	18.86 (6.64)	36–1	170 (49.6)	173 (50.4)
Ignoring the pain	17.37 (6.69)	34–1	190 (55.4)	153 (44.6)
Praying and hoping	23.91 (5.53)	35–10	57 (16.6)	286 (83.4)
Increased behavioral activity	16.55 (7.09)	34–0	246 (71.7)	132 (38.5)

**Table 3:** The relationship between pain coping strategies and perceived social support.

Variable	1	2	3	4	5	6	7	8
Perceived social support	1							

Catastrophizing	*r* = 0.05 *p*=0.416	1						

Reinterpretation of pain	*r* = 0.29 *p* < 0.001	*r* = 0.23 *p* < 0.001	1					

Return attention	*r* = 0.30 *p* < 0.001	*r* = 0.01 *p*=0.852	*r* = 0.61 *p* < 0.001	1				

Talking to oneself	*r* = 0.34 *p* < 0.001	*r* = 0.01 *p*=0.868	*r* = 0.68 *p* < 0.001	*r* = 0.78 *p* < 0.001	1			

Ignoring the pain	*r* = 0.35 *p* < 0.001	*r* = 0.08 *p*=0.174	*r* = 0.72 *p* < 0.001	*r* = 0.80 *p* < 0.001	*r* = 0.80 *p* < 0.001	1		

Increased behavioral activity	*r* = 0.33 *p* < 0.001	*r* = 0.12 *p*=0.033	*r* = 0.75 *p* < 0.001	*r* = 0.77 *p* < 0.001	*r* = 0.74 *p* < 0.001	*r* = 0.80 *p* < 0.001	1	

Praying and hoping	*r* = 0.35 *p* < 0.001	*r* = 0.06 *p*=0.284	*r* = 0.43 *p* < 0.001	*r* = 0.49 *p* < 0.001	*r* = 0.52 *p* < 0.001	*r* = 0.46 *p* < 0.001	*r* = 0.47 *p* < 0.001	1

**Table 4:** The relationship between pain coping strategies and pain acceptance.

Variable	1	2	3	4	5	6	7	8
Acceptance of pain	1							

Catastrophizing	*r* = 0.13 *p*=0.021	1						

Reinterpretation of pain	*r* = 0.27 *p* < 0.001	*r* = 0.23 *p* < 0.001	1					

Return attention	*r* = 0.28 *p* < 0.001	*r* = 0.01 *p*=0.852	*r* = 0.61 *p* < 0.001	1				

Talking to oneself	*r* = 0.31 *p* < 0.001	*r* = 0.01 *p*=0.868	*r* = 0.68 *p* < 0.001	*r* = 0.78 *p* < 0.001	1			

Ignoring the pain	*r* = 0.24 *p* < 0.001	*r* = 0.08 *p*=0.174	*r* = 0.72 *p* < 0.001	*r* = 0.80 *p* < 0.001	*r* = 0.80 *p* < 0.001	1		

Increased behavioral activity	*r* = 0.30 *p* < 0.001	*r* = 0.12 *p*=0.033	*r* = 0.75 *p* < 0.001	*r* = 0.77 *p* < 0.001	*r* = 0.74 *p* < 0.001	*r* = 0.80 *p* < 0.001	1	

Praying and hoping	*r* = 0.32 *p* < 0.001	*r* = 0.06 *p*=0.284	*r* = 0.43 *p* < 0.001	*r* = 0.49 *p* < 0.001	*r* = 0.52 *p* < 0.001	*r* = 0.46 *p* < 0.001	*r* = 0.47 *p* < 0.001	1

**Table 5:** The final (third) regression model of the effect of independent variables on the dependent variable (perceived social support).

Variable	Beta coefficient (*β*)		Standard error	*t* statistic	Significance level (sig)	95% confidence interval	
	Nonstandard	Standard				Lower limit	Upper limit
Fixed coefficient	41.460	—	1.225	33.843	< 0.001	39.050	43.870
Ignoring the pain	0.187	0.225	0.046	4.070	< 0.001	0.097	0.277
Praying and hoping	0.243	0.243	0.055	4.393	< 0.001	0.134	0.352
Living status	−2.869	−0.150	0.938	−3.058	0.002	−4.715	−1.023
Summary of the third model	*p* < 0.001 *F* = 25.634		*R*-square = 0.185		Adjusted-*R*-square = 0.178		

**Table 6:** The final (fourth) regression model of the effect of independent variables on the dependent variable (pain acceptance).

Variable	Beta coefficient (*β*)		Standard error	*t* statistic	Significance level (sig)	95% confidence interval	
	Nonstandard	Standard				Lower limit	Upper limit
Fixed coefficient	17.375	—	1.257	13.828	< 0.001	14.903	19.847
Praying and hoping	0.175	0.223	0.044	3.989	< 0.001	0.089	0.261
Increased behavioral activity	0.113	0.191	0.034	3.362	0.001	0.047	0.180
Catastrophizing	−0.112	−0.170	0.033	−3.411	0.001	−0.177	−0.048
Number of pain sites	−0.600	−0.137	0.220	−2.728	0.007	−1.033	−0.167
Summary of the fourth model	*p* < 0.001 *F* = 17.707		*R*-square = 0.173		Adjusted-*R*-square = 0.163		

## Data Availability

The data that support the findings of this study are available on request from the corresponding author. The data are not publicly available due to privacy or ethical restrictions.
